# A New Multistage Medical Segmentation Method Based on Superpixel and Fuzzy Clustering

**DOI:** 10.1155/2014/747549

**Published:** 2014-03-09

**Authors:** Shiyong Ji, Benzheng Wei, Zhen Yu, Gongping Yang, Yilong Yin

**Affiliations:** ^1^School of Computer Science and Technology, Shandong University, Jinan 250101, China; ^2^College of Science and Technology, Shandong University of Traditional Chinese Medicine, Jinan 250355, China

## Abstract

The medical image segmentation is the key approach of image processing for brain MRI images. However, due to the visual complex appearance of image structures and the imaging characteristic, it is still challenging to automatically segment brain MRI image. A new multi-stage segmentation method based on superpixel and fuzzy clustering (MSFCM) is proposed to achieve the good brain MRI segmentation results. The MSFCM utilizes the superpixels as the clustering objects instead of pixels, and it can increase the clustering granularity and overcome the influence of noise and bias effectively. In the first stage, the MRI image is parsed into several atomic areas, namely, superpixels, and a further parsing step is adopted for the areas with bigger gray variance over setting threshold. Subsequently, designed fuzzy clustering is carried out to the fuzzy membership of each superpixel, and an iterative broadcast method based on the Butterworth function is used to redefine their classifications. Finally, the segmented image is achieved by merging the superpixels which have the same classification label. The simulated brain database from BrainWeb site is used in the experiments, and the experimental results demonstrate that MSFCM method outperforms the traditional FCM algorithm in terms of segmentation accuracy and stability for MRI image.

## 1. Introduction 

The medical image is human body image captured by medical imaging equipment, including Computed Tomography (CT), Magnetic Resonance imaging (MRI), and Ultrasonography (US.) Based on the computer graphics technology, the quality and displaying method of medical image have greatly improved. MRI technology has many advantages such as nonradioactive contamination, high resolution, without electricity radiation damage to the human body, so it is widely applied in clinical diagnosis and treatment now. MRI image processing promotes the development of medical research and has important applications value. According to the above process, the accurate medical image segmentation by computational techniques plays an essential role [1].

In MRI medical image segmentation, the image is parsed into a number of meaningful regions based on the consistency principle. These regions usually do not cross each other and satisfy the consistency principle. If merging any two adjacent regions, it will break this principle. Hence, the medical image segmentation can be seen as the classification of image pixels in this viewpoint.

**Figure 1:**
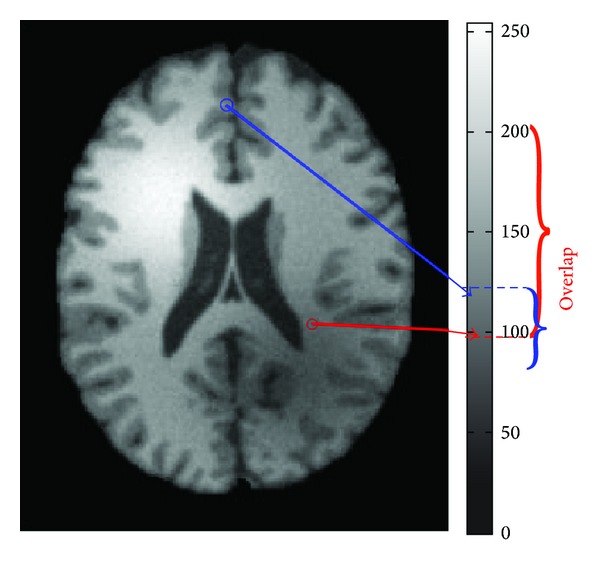
The illustration of bias field in MRI image.

As is shown in Figure 1, the region marked by blue circle represents gray matter and region marked by red circle represents white matter. Generally, white matter has a larger gray value than gray matter. Nevertheless, with the influence of intensity inhomogeneity, gray matter in blue circle has a larger gray value than white matter in red circle, which makes an overlap in the image.

The influence is that a slowly varying shading artifact over the MRI image can produce errors with conventional intensity-based classification. Consequently, several methods for MRI intensity inhomogeneity correction are applied before the image segmentation. Series methods on intensity inhomogeneity correction/removal have been proposed in the last two decades [2–5]. Nevertheless, intensity inhomogeneity correction is still incompletely solved problem. Because of this and the evolving MRI technology and associated applications, the problem of intensity inhomogeneity correction will certainly continue to be paid more research attention in the future [6].

In previous works of MRI image segmentation, as the thorough application of statistical theory, fuzzy set theory and machine learning theory deserve paying much more attention [3, 7–11]. In particular, for the fuzziness of the medical image, the fuzzy theory is introduced into the medical image processing, which generates lots of new segmentation methods and achieves good segmentation results. The most representative method is the fuzzy C-means clustering algorithm proposed by Bezdek et al. in [12]. Pham and Prince proposed the adaptive fuzzy segmentation method to segment the MRI images [11]. In order to solve the bias field estimation and segmentation problem, Ahmed et al. in [5] proposed the modified fuzzy C-means algorithm for the MRI image segmentation. Amini et al. in [13] made use of the FCM (fuzzy C-means) to solve the segmentation problem of the thalamus in the brain MRI image. Shen et al. in [14] modified the fuzzy C-means clustering method to segment the brain MRI image. Awate et al. in [15] proposed a segmentation framework to deal with the DT and MRI image based on the fuzzy and the nonparametric estimation. Halt et al. in [16] proposed the Bayesian segmentation method based on the local adaptive fuzzy, which was used to solve the volume measurement problem in the PET image. The improved FCM method based on the histogram was proposed in [17] by Zhang et al. for the medical image segmentation.

The FCM algorithm has many advantages such as being without supervision, simple realization, and fast processing speed, which can carry out the accurate segmentation for the image with high contrast and signal-to-noise ratio. But there are also lots of obvious disadvantages. In the process of fuzzy clustering, the gray value distance between single pixel and cluster center can be considered only, while the influence of the adjacent pixels is neglected. That is to say, the spatial information cannot be well used in image segmentation. So the large deviation will be produced when the FCM algorithm is used to* segment* the brain MRI image with noises and the low signal-to-noise ratio [18]. Moreover, in order to segment the image with intensity inhomogeneity, the result by the FCM algorithm will be unsatisfactory because of the low contrast in the whole image.

Superpixels, also known as regions in an over segmentation of the image, would be more natural and presumably lead to more efficient processing [19]. The superpixel method had been increasingly used in image processing field, which can group pixels using the degree of feature similarity between pixels and acquire the redundant information of the image. Hence, it can greatly reduce the complexity of image post-processing tasks. The method that combines the superpixel and the clustering has successfully solved the arterial segmentation and tracking problem in the CT image [20]; Zhang and Ji in [21] proposed an image segmentation framework based on the superpixel and had achieved good result on natural images; Gan et al. applied the superpixel to the multiclass segmentation of the SAR image [22]; Zhou et al. in [23] proposed a superpixel driver method to track the target; Gong and Liu have employed the superpixel method to detect the rock in the image in [24]; the superpixel method has been used to perform segmentation of the background in the image by Jiang in [25]; Li et al. had proved that the particle aggregation information provided by the superpixel is useful for image segmentation in [26].

The superpixel method can make full use of spatial information, this feature makes the algorithm has well anti-noise performance. Besides, it can save the edge information of the original image during the process of enhancing the local consistency. The segmented atomic area has some image characteristics such as shape boundary contour information and area histogram and, in which characteristics are not affiliated with the single pixel. So the superpixel method can improve the segmentation accuracy and the processing time. In addition, the gray value of each pixel within the superpixels is very similar, and the phenomenon of intensity inhomogeneous will not exit in the superpixels.

Due to the fuzziness feature, it is hard to segment MRI images well. FCM algorithm may solve this problem to a certain extent, but it is sensitive to noise and bias field. To take advantages of superpixel method to reduce the effect of these problems, a new multistage segmentation method based on the superpixel and designed FCM is proposed, which does not only make use of the beneficial aspects of the fuzzy clustering algorithm in the medical image, but also use the superpixel method to enhance the space constraint and it effectively solved the inhomogeneity problem.

The structure of this work is as follows. In Section 2, the basics of superpixel method and FCM algorithm are presented. In Section 3, the proposed MCFCM method is introduced, along with detailed multistage segmentation processing. The experimental results are discussed in Section 4, and from the numerical analysis, conclusions are presented in Section 5.

## 2. Preliminaries

In this section, the fundamentals of the superpixel method and the FCM algorithm are explained in detail.

### 2.1. Superpixel Level Segmentation Method

The superpixel is to use some algorithms to aggregate some pixels together to form atomic regions that have a certain meaningful perception. Atomic regions are used to replace the regional grids that are segmented rigidly. The superpixel as the basic unit seems inefficient. What leads to this situation is that it needs to carry out a task completely unrelated to the final decision task to converge pixels into different groups. However, redundant information can be got partially from the data and the risk of merging unrelated pixels in the process of aggregating pixels into superpixels can be minimized, which can help to achieve the purpose of decision making. Meanwhile, superpixel helps to obtain some characteristics of the statistical information in a natural adaptive area rather than an artificial divided area. As boundary information is considered when the image is segmented into superpixels, more accurate segmentation results can be got by finding some superpixels belonging to the target.

There are many superpixel segmentation methods in recent years, such as turbo pixel [27, 28], normalized cuts [29], quick shift [30], and SLIC superpixel [19]. The feature of normalized cuts segmentation is that the number of superpixels can be controlled, the shape of superpixel is relatively compact, and the area of superpixel is broadly similar as well. But normalized cuts segmentation has a low running speed, especially for large pictures that need large amount of computation. SLIC is an efficient method that uses color similarity of pixels and spatial information of image to generate compact and uniform superpixels. Due to that superpixels achieved by normalized cuts and SLIC are always compact and with uniform shape, their semantics performance is poor. Superpixels with compact structure cannot cover a complete object, and uniform shapes lead to different semantic levels in segmenting target of different scales. Quick shift is a gradient based pattern search segmentation method. This method achieves image segmentation by promoting data points in feature space move along the Parzen density ascendant direction. Quick shift algorithm cannot limit the shape and size of superpixel, and the compactness of superpixel is also poor. Turbo pixel algorithm can control the number of superpixels and has a high processing speed. What is more, superpixels generated by turbo pixel have approximate sizes, and the boundaries are more close to the real image. The basic idea of turbo pixel is to select a certain number of seed points on the image and devise a flow by which curves evolve to obtain superpixel boundaries.

In this paper, the turbo pixel method is utilized, and the details of this method are presented as follows.

Let *C* be a vector of curve coordinates parameterized by *t*, a parameter to denote evolution in time. Let *N*represent its outward normal and let each point move with speed *S*. Then let level set curve evolution equation be

(1) $$\begin{array}{c} \frac{\partial C}{\partial t}=SN. \end{array}$$

This curve evolution equation is implemented by first embedding *C* as a level set of a smooth and continuous function Ψ : *R* ^2^ × [0, *τ*) → *R* ^2^ and then evolving this embedding function according to

(2) $$\begin{array}{c} \psi_{t}=-S||\nabla \psi ||. \end{array}$$

And the first-order discretization formula of (2) is

(3) $$\begin{array}{c} \psi^{n+1}=\psi^{n}-S_{I}S_{B}||\nabla \psi^{n}||\Delta t. \end{array}$$

Each application of formula (2) corresponds to one “time step” Δ*t* in the evolution of the boundary. The key term controlling the evolution is the product of two speeds *S*_*I*_ *S*_*B*_. *S*_*I*_ depends on local image structure and superpixel geometry at each boundary point and *S*_*B*_ depends on the boundary point's proximity to other superpixels. The *S*_*I*_ of formula (3) consists of reaction-diffusion-based shape segmentation model and the geodesic active contour model as

(4) SI(x,y)=[1−ακ(x,y)]φ(x,y) −β[N(x,y)·∇φ(x,y)].

The first half of the formula (4) that named reaction-diffusion term ensures that the boundary's evolution slows down when it gets close to a high gradient region in the image. *φ*(*x*, *y*) is local affinity function: *φ*(*x*, *y*) = *e* ^−*E*(*x*,*y*)/*v*^, *E*(*x*, *y*) = ||∇_*I*_||/(*G*_*σ*_ · ||∇_*I*_|| + *γ*), computed for every pixel on the image plane, and the *φ*(*x*, *y*) has a low value near the edge and has a high value elsewhere. *κ* = (Ψ_*xx*_Ψ_*y*_^2^ − 2Ψ_*x*_Ψ_*y*_Ψ_*xy*_ + Ψ_*yy*_Ψ_*x*_^2^)/(Ψ_*x*_^2^ + Ψ_*y*_^2^)^3/2^ expresses the curvature of the boundary at point (*x*, *y*) and smoothes the evolving boundaryand *α* is balancing parameter that weighs the contribution of the curvature term. Intuitively, the latter part of the formula (4) that named doublet term ensures that the boundary is attracted to image edges *N* = ∇Ψ/||∇Ψ||.

The entire algorithm of turbo pixel is summarized as follows.

**Step 1**

Initialize seeds, and perturb the seed positions away from high gradient regions.

**Step 2**

Set all seed pixels to “assigned.”

**Step 3**

Set Ψ^0^ to be the signed Euclidean distance from the “assigned” regions,

∑_*x*,*y*_[Ψ^0^(*x*, *y*) ≥ 0]→ assigned pixels.

**Step 4**

Compute *φ*(*x*, *y*), *n* → 0.

**Step 5**

While change in assigned pixels is large, do

- compute *S*_*I*_ *S*_*B*_
- *S*_*I*_ *S*_*B*_ → *S*, extend the speed *S* in a narrow band near the zero level-set of Ψ^*n*^;
- compute Ψ^*n*+1^ by evolving Ψ^*n*^ within the narrow band, *n* = *n* + 1;
- ∑_*x*,*y*_[Ψ^*n*^(*x*, *y*) ≥ 0]→assigned pixels;
- Homotopic skeleton of Ψ^*n*^ → *B*.

**Step 6**

Return superpixel boundary *B*.

**Figure 2:**
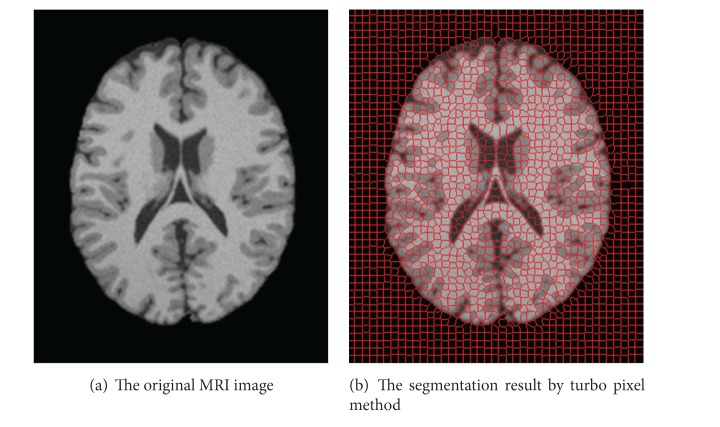
An example of the superpixel level segmentation results.

The following Figure 2 shows the superpixel segmentation results of an MRI image by turbo pixel method.

### 2.2. FCM Algorithm

FCM algorithm was proposed by Dunn and later on modified by Bezdek [12]. The basic principle of FCM is the iterative minimization of the following objection function:

(5) $$\begin{array}{c} J=\overset{c}{\underset{i=1\;\;}{\sum }}\overset{N}{\underset{k=1}{\sum }}u_{ik}^{p}{||y_{k}-v_{i}||}^{2}. \end{array}$$

Let {*y*_*k*_, *k* = 1,2,…, *N*} denote an image with *N* pixels to be categorized into *c* clusters, {*v*_*i*_, *i* = 1,2,…, *c*}denote every cluster centers, and *C* = (*c*_1_, *c*_2_,…, *c*_*c*_) is the cluster center matrix. The vector *U*_*k*_ = (*u*_1*k*_, *u*_2*k*_,…, *u*_*ik*_)^*T*^ denotes the membership of the *k*th pixel in *i* clusters, *u*_*ik*_  (*u*_*ik*_ ∈ [0,1]) is the membership of the *k*th pixel in the *i*th cluster, the *U* = (*U*_1_, *U*_2_,…, *U*_*k*_) is the membership matrix, *p* is the membership function index that controls the fuzziness of resulting partitions, and ||*y*_*k*_−*v*_*i*_||^2^ is a norm metric which usually uses Euclidean distance.

The algorithm steps are as follows.

**Step 1**

Set iteration stop threshold *ε*, initialize the membership matrix *U* and cluster center matrix *C*, and let iteration counter *q* be equal to 0.

**Step 2**

The membership function is updated as

(6) $$\begin{array}{c} u_{ik}=\frac{{\left({1/||y_{k}-v_{i}||}^{2}\right)}^{1/(p-1)\;}}{\sum_{j=1}^{c}{\left(1/{||y_{k}-v_{j}||}^{2}\right)}^{1/(p-1)}}. \end{array}$$

**Step 3**

The cluster centers are updated as

(7) $$\begin{array}{c} v_{i}=\frac{\sum_{k=1}^{N}u_{ik}^{p}y_{k}}{\sum_{k=1}^{N}u_{ik}^{p}}. \end{array}$$

**Step 4**

When the objective function value changes less than the setting threshold, then stop the algorithm.

## 3. The Proposed MSFCM Method

The Multistage medical image segmentation method Based on superpixel and Fuzzy clustering (MSFCM) is proposed taken into account the advantages of fuzzy clustering in medical image processing and superpixel's advantages in strengthening space information and effectively processing in intensity inhomogeneity problem. The MSFCM is based on superpixel, which can make up the insufficiencies in noise and bias field processing aspect by only using original FCM. And this method can be divided into three stages.

- *Rough Segmentation*. Partition the image into superpixels.
- *Deep Segmentation*. Parsing superpixels which have large variance into smaller atomic regions.
- *Cluster and Label Superpixels*. Cluster superpixels with FCM method and label superpixel to the appropriate class using spatial and gray information and finally obtain the segmentation result by merging superpixels belonging to the same class.

**Figure 3:**
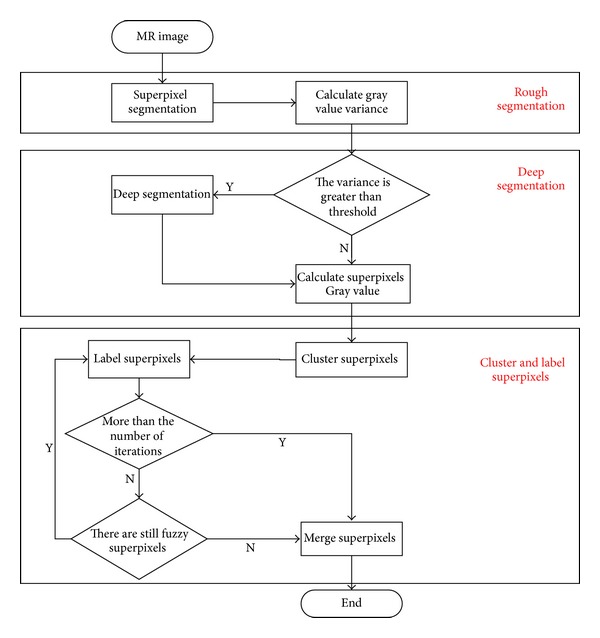
The flowchart of MSFCM.

The flowchart is shown in Figure 3 and the detailed process of MSFCM is shown as follows.

### 3.1. Rough Segmentation

Superpixel segmentation is equivalent to an image over segmentation, and its essence is also described in the form of image segmentation. So it is suitable to preprocess the image with superpixel method to get the rough segmentation of the MRI image. For an MRI image which has a size of M*N  (0 ≤ *x* ≤ *M*, 0 ≤ *y* ≤ *N*), let Λ(*x*, *y*) represent the entire image grid. The segmentation for Λ can be considered as dividing it into *n* nonempty region (*R*_1_, *R*_2_,…, *R*_*n*_) which must satisfy the following five conditions.

- ∪_*i*=1_^*N*^ *R*_*i*_ = Λ.
- For all *i* and *j*, when *i* ≠ *j*, *R*_*i*_∩*R*_*j*_ = *ϕ*.
- For all *R*_*i*_, *i* = 1,2,…, *N*, *P*(*R*_*i*_) = true.
- For all *i* and *j*, when *i* ≠ *j*, *P*(*R*_*i*_∩*R*_*j*_) = false.
- For all *R*_*i*_, *i* = 1,2,…, *N*,  *R*_*i*_ is a connected region.

*P*(*R*_*i*_) is the logical predicate of the elements for every *R*_*i*_  (*i* = 1,2,…, *N*) and *ϕ* represent the empty set.

MSFCM used turbo pixel method to segment the image. And  *L* superpixels *R*_*i*_  {*i* = 1,2,…, *L*} can be got as the rough segmentation result.

### 3.2. Deep Segmentation

As part of the boundary of some regions in the MRI image is fuzzy, a problem can occur after superpixel level segmentation, which is that the different tissues are wrongly divided into the same superpixel. In order to reduce such errors, it is necessary to deeply segment the image on some specific superpixels.

Because such superpixel's variance is bigger than other superpixels, it is feasible to take the sequence of the front of a certain percentage of superpixels to do the further segmentation. Automatic threshold segmentation method can solve this problem well. Furthermore, a scale parameter *t* (the value of *t* can be set according to specific situation) is introduced to eliminate the impact of noise points and those regions in which proportion in original superpixel is greater than *t* after threshold segmentation is saved to the deep segmentation process.

After this procession, *K*  (*K* ≥ *L*) superpixels *R*_*i*_  {*i* = 1,2,…, *k*} can be got, and these regions are the objects for FCM clustering.

**Figure 4:**
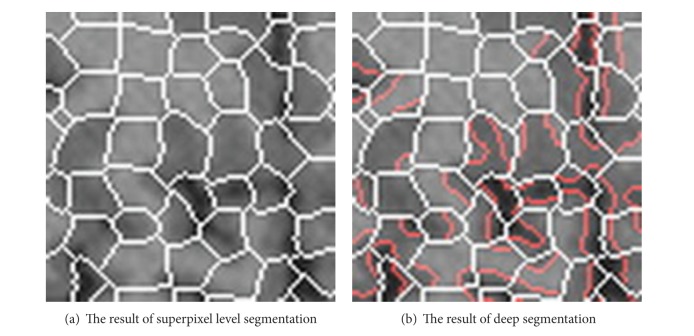
An example of the typical deep segmentation result.

An example of the typical deep segmentation result is shown in Figure 4, the white boundary is generated by superpixel method, and the red boundary is generated by deep segmentation process. It is easy to see that different tissues can be separated clearly after deep segmentation.

### 3.3. Cluster and Label Superpixels

To obtain the final result, it is essential to cluster and label the superpixels to the right classifications. And this process can be divided into the following three parts.

#### 3.3.1. Part 1: FCM Clustering

As far as MRI image is concerned, it can be divided into three tissues: the gray matter, white matter, and cerebrospinal fluid. So the classification parameter of FCM could be set to three. In this paper, the mean of every superpixel's gray value *μ* is used as clustering parameter. As the FCM clusters MRI image to *K* superpixels generated in Section 3.2, the clustering center matrix *C*(*c*_1_, *c*_2_, *c*_3_) and membership matrix *U* can be obtained. And each superpixel's classification label is determined by *U*.

#### 3.3.2. Part 2: Label the Superpixels

In view of the fuzzy and inhomogeneity property of brain MRI medical image, it is not feasible to label the superpixels to the right classification with clustering results directly. So it is necessary to introduce other information to help label superpixels.

As the organization of brain MRI image has the characteristics of continuity, spatial adjacent information of superpixel is presented to determine which class the superpixel belongs to. Let *S*(*s*_1_, *s*_2_,…, *s*_*n*_) indicate the similarity between adjacent superpixels. *s*_*i*_ is the similarity value between current superpixel and its *i*th adjacent superpixels.

To measure the similarity between superpixels, it is necessary to employ a function which has characteristic as follows.

- For superpixels that have small gray-scale difference, they should return a large value in similarity.
- For superpixels that have large gray-scale difference, they should return a small value in similarity.
- When the gray-scale difference between two superpixels exceeds a certain threshold, the similarity should decrease rapidly.

Based on the above requirements, Butterworth function is employed in this paper. The Butterworth function form is as follows:

(8) $$\begin{array}{c} S_{i}=\frac{1}{1+{\left(\left(\mu -\mu_{i}\right)/\eta \right)}^{n}}. \end{array}$$

**Figure 5:**
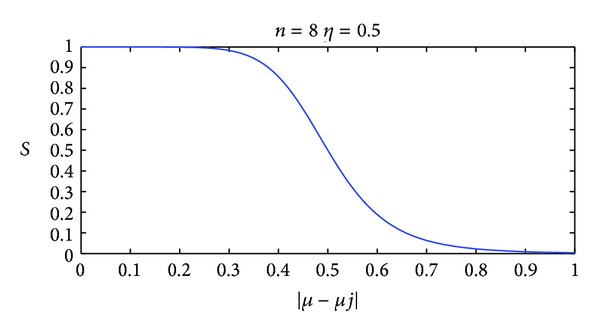
An example of the typical Butterworth function curve.

In (8), *η* is the tolerance value. With the increase of *η*, gray value difference would be allowed more larger as judging the similar superpixels; *μ* is the mean gray value of the superpixel to be judged, and *μ*_*i*_ is the mean gray value of this superpixel's adjacent superpixel *I*; *n* is function series; the greater of *n* value, the faster function declines. The Butterworth function curve is shown in Figure 5.

In order to label the superpixels to the right classification, a broadcast method based on spatial adjacent information is introduced. The detailed steps are presented as follows.

**Step 1**

For superpixel *R*_*i*_, define the membership vector *U*_*i*_(*u*_1_, *u*_2_, *u*_3_), if there exist *u*_*i*_ = max⁡⁡{*u*_1_, *u*_2_, *u*_3_} > *T*_*c*_ (*T*_*c*_ is confidence threshold), then this superpixel is marked to *i*th classification, else the superpixel should be marked to fuzzy block which is denoted by *F*.

**Step 2**

For *R*_*j*_ ∈ *F*, assuming that its adjacent superpixels set is *Ω* = {*R*_*j*1_, *R*_*j*2_,…, *R*_*jk*_}, compute *S*_*j*_(*s*_*j*1_, *s*_*j*2_,…, *s*_*jk*_) of *R*_*j*_ with each element of  *Ω* respectively.

**Step 3**

If there exists a *s*_*ij*_ = max⁡*S*_*j*_ > *T*_*s*_, then the *R*_*j*_ was marked to the same classification with *R*_*ji*_. *T*_*s*_ is the confidence threshold.

**Step 4**

If the number of iterations is not more than a limited number and there are still fuzzy blocks, then go to Step 2.

**Step 5**

If the number of iterations is more than a limited number and there are still fuzzy blocks, then for fuzzy block *R*_*i*_ which has a membership vector *U*_*i*_ = (*u*_1_, *u*_2_, *u*_3_), if *u*_*j*_ has the maximum value in *U*_*i*_, *R*_*i*_ should be marked to *j*th classification.

After all above steps, each superpixel has a clear classification.

#### 3.3.3. Part 3: Merge Superpixels

Superpixels are going to be merged after processing Part 2. Superpixels which belong to the same classification and adjacent to each other will be merged, and the final segmentation result will be achieved.

**Figure 6:**
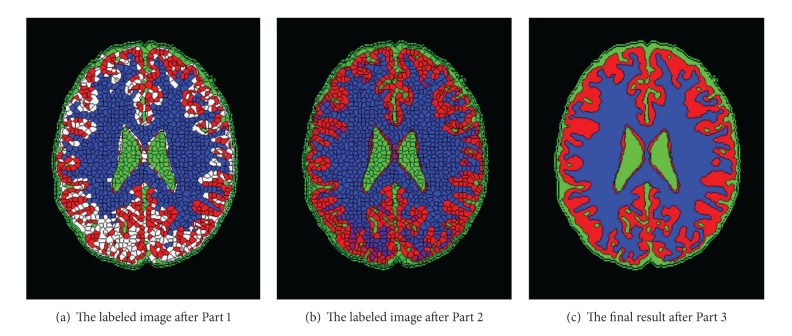
An example of the labeling classification for superpixels.

Figure 6 shows the change of the superpixels' classification in this stage. In the figure, blue region represents the white matter, red region represents the gray matter, green region represents cerebrospinal fluid, and white region represents the fuzzy block. As is shown in Figure 6(a), there are lots of fuzzy blocks with the influence of inhomogeneity property and noise, while after being adopted by the broadcast label method proposed in this paper, the fuzzy blocks can be labeled to the proper classification and the final result can be obtained by merging the superpixels with the same classification which is shown in Figure 6(c).

## 4. Experimental Results

To verify the effectiveness of the algorithm, the synthetic MRI images with ground truth from Brain Web [31] are used as experimental data. In the database, noise parameters are settled as 0%, 3%, 5%, 7%, and 9%, and bias field parameters are settled as 0%, 20%, and 40%, and then 30 images are selected for each image sequence. Therefore, a total of 15 experiments need to be done. In these experiments, Jaccard similarity (JS) is applied as the metric to quantitatively evaluate the segmentation accuracy. The JS is defined as

(9) $$\begin{array}{c} J\left(S_{1},S_{2}\right)=\frac{\left|S_{1}\cap S_{2}\right|}{\left|S_{1}\cup S_{2}\right|}. \end{array}$$

*S*_1_, *S*_2_ represent segmentation results of different algorithms and ground truth, respectively.

**Figure 7:**
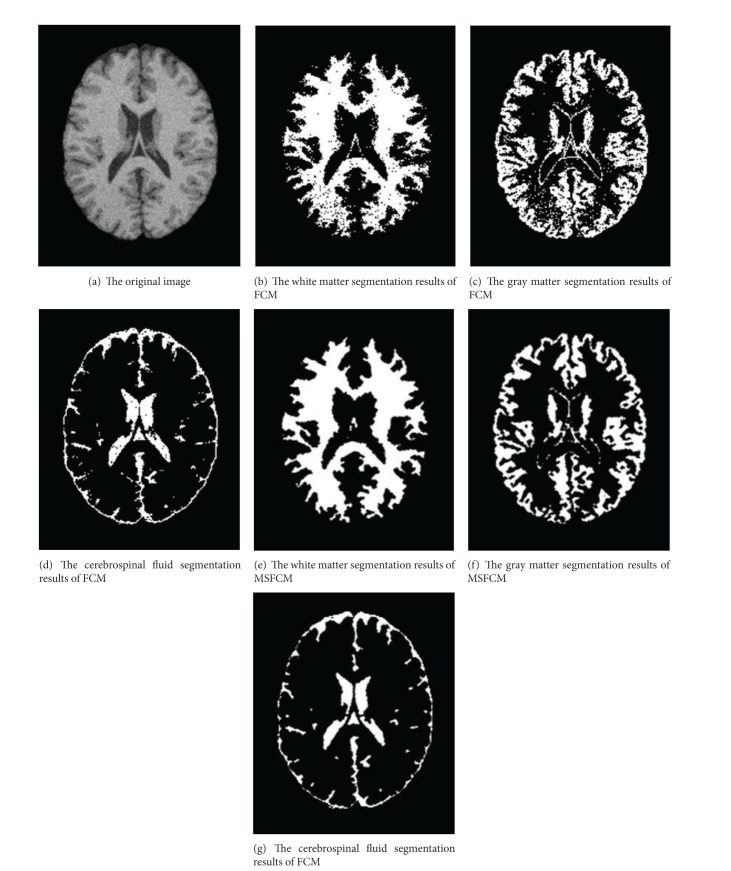
A typical comparison of FCM and MSFCM segmentation results.

**Figure 8:**
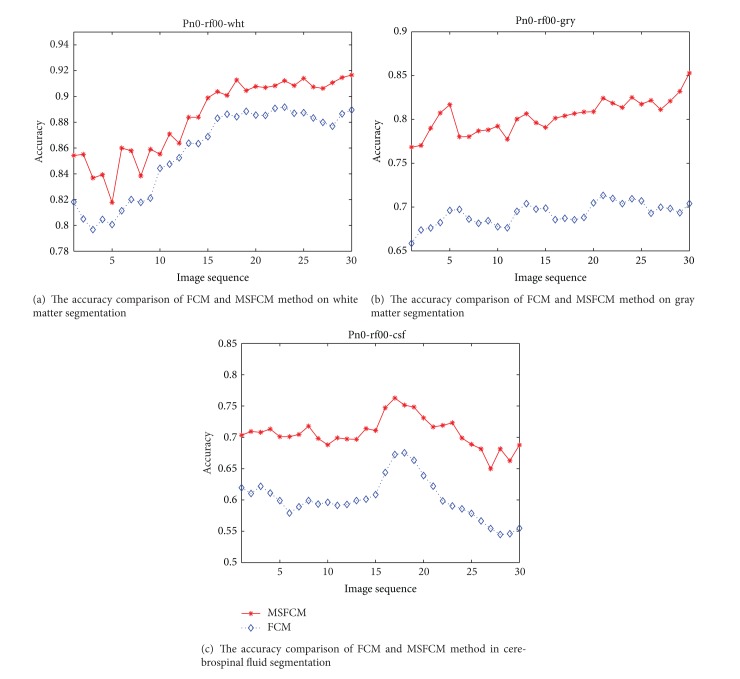
The comparison of segmentation accuracy as the 0% noise and 0% bias field.

**Figure 9:**
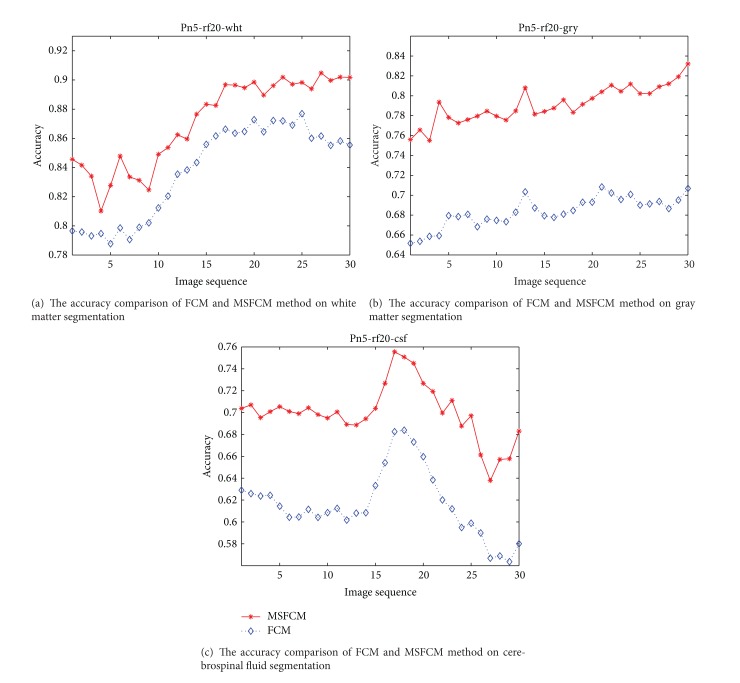
The comparison of segmentation accuracy as 5% noise and 20% bias field.

**Figure 10:**
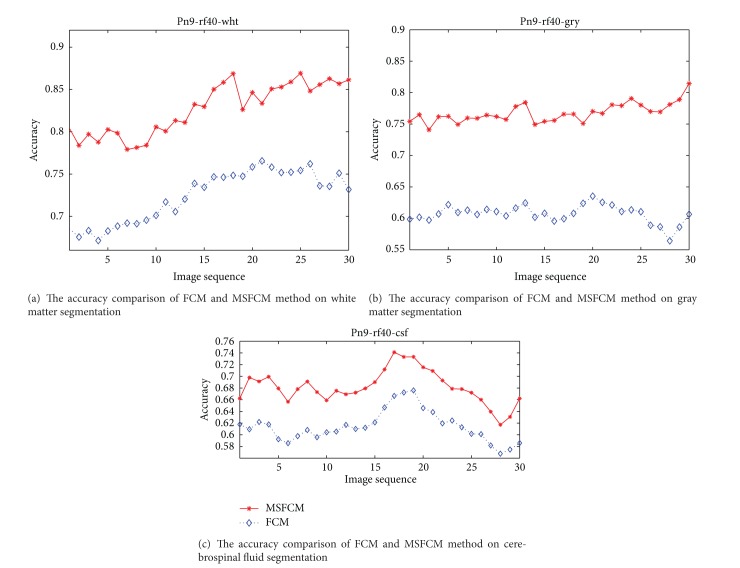
The comparison of segmentation accuracy as 9% noise and 40% bias field.

Under the setting 7% noise and 20% bias field, one of the segmentation results of MSFCM and FCM is shown in Figure 7. The MRI segmentation accuracy comparison of the proposed method MSFCM and the FCM is shown in Figures 8, 9, and 10 under the different setting noise and bias field.

Table 1 is the mean accuracy table of 30 images under the different setting noise parameter when bias field parameter is fixed. The mean accuracy table of 30 images under the different setting bias field parameter as the settled noise parameter is shown in Table 2.

**Figure 11:**
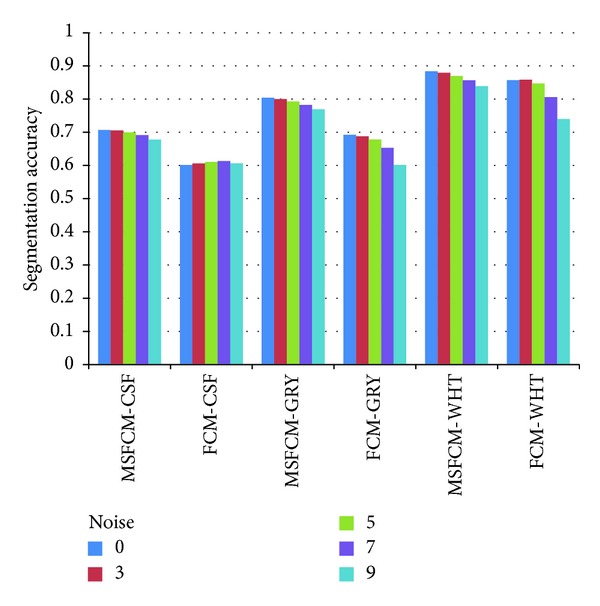
The robustness comparison of MSFCM and FCM.

The 15 comparative experiments results in the various conditions show that the segmentation accuracy of MSFCM is much higher than FCM. In addition, in the case of gradually increase of noise and bias field parameter, the MSFCM's accuracy rate of decline is far less than FCM. As shown in Figure 11, with the increase of noise, the accuracy rate of MSFCM decrease more slowly than FCM in white matter and gray matter. The result shows that the proposed algorithm has advantages in accuracy and robustness compared with FCM.

Due to that the clustering objects of MSFCM algorithm are the superpixels instead of pixels, this transformation increases the granularity of the clustering and is able to make full use of spatial constraint information, so MSFCM method has a good performance in terms of noise immunity. Furthermore, superpixels, which can also be considered as atomic regions, have some perceived significance: superpixel has lower difference on gray value in its internal space, and this feature can reduce the impact of inhomogeneity in the whole image. So in processing an image which has bias fields, the proposed method can effectively avoid the impact of this phenomenon on the segmentation.

In superpixel clustering processing, MSFCM utilizes Butterworth function to process class discrimination issues for the fuzzy blocks. This way could take advantage of adjacent superpixels information and improved accuracy class determination compared with rigid partition.

MSFCM combined superpixel method and FCM method, effectively used both advantages in image processing, and targeted to overcome FCM's defects in noise and bias field aspect.

Therefore, MSFCM algorithm has higher accuracy and higher robustness in segmentation than FCM algorithm.

## 5. Conclusion and Discussion

In this paper, the MSFCM algorithm is presented to segment the brain MRI image, which consists of the superpixel method and the FCM algorithm. The image was firstly parsed into several superpixels, and then deep segmentation is to be done for the areas with bigger gray variance than setting threshold. And to get the fuzzy membership of each superpixel, the FCM algorithm is used to cluster the superpixels rather than pixels, and the membership is used to determine the classification for these superpixels. Finally, the segmented brain MRI image is achieved by merging the superpixels with the same classification.

The experiments reveal that the proposed method is more efficient and stable than FCM, and has achieved good results in segmenting MRI images with noise and intensity inhomogeneity. This advantage made it possible to obtain a high accuracy and effectiveness in the human brain MRI image segmentations compared to those outlined by the experts and by the FCM method according to the evidence of similarity metrics. Additionally, the experimental results have also shown that the local exploitation of broadcast method to properly label superpixels classifications and the Butterworth function to measure the similarity between superpixels are highly suitable for medical image applications, including segmenting datasets of sequential medical images within an appropriate computational time.

## Highlight

1. A broadcast method taking advantage of spatial adjacent information is proposed to label superpixels to the proper classification, which improves the accuracy compared with the inflexible label method.
2. The Butterworth function is introduced and designed to measure the similarity between superpixels.
3. The MSFCM is a new multistage segmentation method based on the superpixel method and the FCM algorithm, which combine the advantages of the two methods to solve the influence of noise and the bias filed in the brain MRI medical image segmentation effectively and robustly.

## Figures and Tables

**Table 1:** The mean accuracy of 30 images under different setting noise parameter as 0% bias field.

Method	Noise				
	0	3	5	7	9
MSFCM-CSF	0.7071	0.7058	0.6999	0.6918	0.6781
FCM-CSF	0.6016	0.6064	0.6110	0.6134	0.6067
MSFCM-GRY	0.8039	0.8003	0.7930	0.7825	0.7696
FCM-GRY	0.6923	0.6880	0.6789	0.6529	0.6018
MSFCM-WHT	0.8837	0.8793	0.8697	0.8566	0.8390
FCM-WHT	0.8574	0.8586	0.8473	0.8065	0.7404

**Table 2:** The mean accuracy of 30 images under different setting bias field parameter as 0% noise.

Method	Bias-filed		
	0	20	40
MSFCM-CSF	0.7071	0.7101	0.7073
FCM-CSF	0.6016	0.6075	0.6187
MSFCM-GRY	0.8039	0.8029	0.8016
FCM-GRY	0.6923	0.6963	0.7049
MSFCM-WHT	0.8837	0.8839	0.8787
FCM-WHT	0.8574	0.8517	0.8345
